# Metastatic Mons Pubis Soft Tissue Mass

**DOI:** 10.7759/cureus.34277

**Published:** 2023-01-27

**Authors:** Nadezda Buntic, Taylor Brown, Muhammad Raafey, Christopher Yeary

**Affiliations:** 1 Research, Lincoln Memorial University-DeBusk College of Osteopathic Medicine, Harrogate, USA; 2 Internal Medicine, Norton Community Hospital, Norton, USA; 3 Pathology, East Tennessee State University, Johnson City, USA; 4 General Surgery, Norton Community Hospital, Norton, USA

**Keywords:** cutaneous metastasis, gynecological cancer, endometrial adenocarcinoma, endometrial cancer, cutaneous mass

## Abstract

Cutaneous spread of solid malignancies is rare. We present the case of a 61-year-old woman with a history of endometrial adenocarcinoma, presenting two years after a total abdominal hysterectomy with bilateral salpingo-oophorectomy with a mass on her mons pubis. The mass was found to be an adenocarcinoma favoring a gynecological origin.

## Introduction

With the exception of breast cancer, the cutaneous spread of solid malignancies is very rare. The general incidence of an internal malignancy spreading cutaneously has been reported anywhere from 0.7% to 10% [1]. The reported prevalence of endometrial cancer metastasizing to the skin is 0.8% [2]. We are presenting a rare case of a 61-year-old female with a history of well-differentiated International Federation of Gynecology and Obstetrics (FIGO) grade 1 endometrioid adenocarcinoma who had a large soft tissue lesion that was found to be a cutaneous metastasis.

## Case presentation

A 61-year-old woman with a known history of well-differentiated FIGO grade 1 endometrioid adenocarcinoma presented to the clinic with complaints of a lesion on her mons pubis. She stated that there had been swelling that came and went for a few months, but the current 13-cm lesion had been constant for a month. She had been treated by her primary care provider with oral amoxicillin-clavulanate 125 mg twice a day for a week prior to referral to general surgery. Two years prior to her presentation, she had an open total abdominal hysterectomy and bilateral salpingo-oophorectomy (TAH with BSO). A lymphadenectomy was not performed at that time due to her large body habitus, with a BMI of 39. Following her TAH with BSO, she received 25 treatments of adjuvant pelvic radiotherapy utilizing a four-field box technique to the pelvis and regional lymph nodes. The vaginal cuff received high-dose radiation to decrease the likelihood of recurrence.

**Figure 1:**
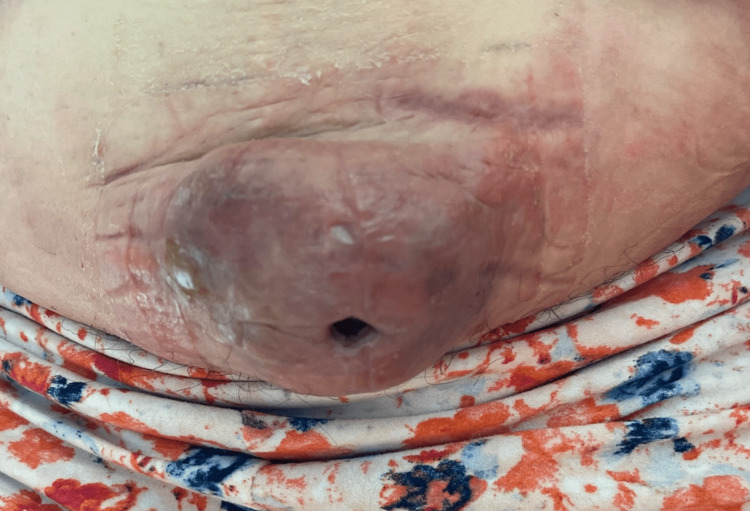
Gross photo of the mass.

The new lesion was 13 cm in diameter, raised, reddish-purple, and indurated, with scant purulent drainage (Figure 1). 

**Figure 2:**
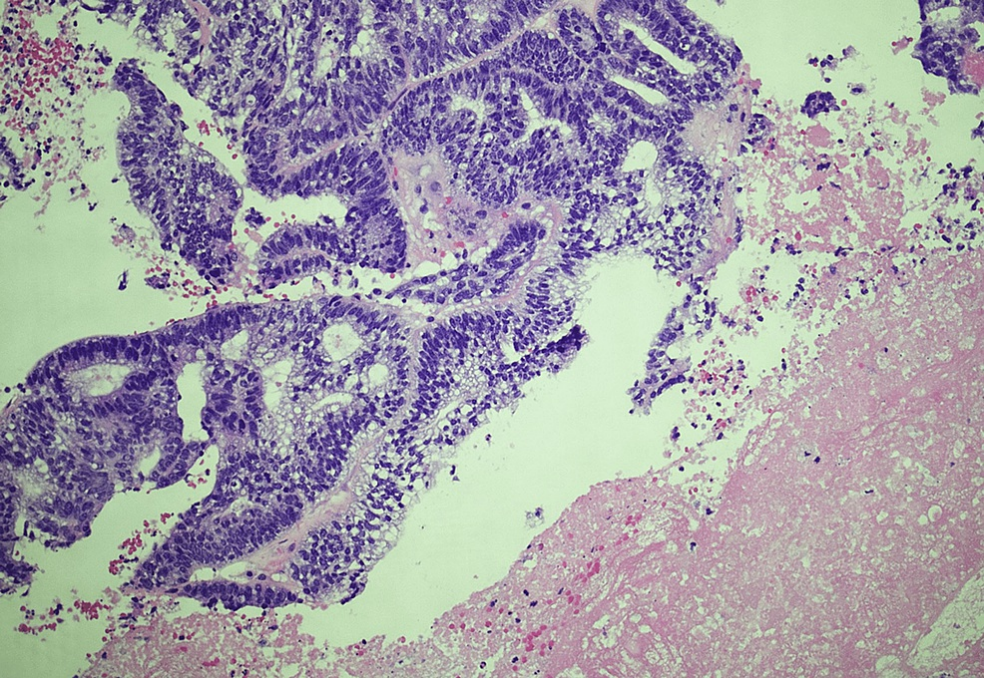
Histology slide of the mass. Malignant glands are present in a background of necrosis.

Given the suspicious appearance of the lesion, further imaging was ordered, and an incision and drainage with core biopsy were performed. A non-contrasted pelvic CT scan showed no signs of adenopathy or invasive depth to the lesion and showed the mass to be 13 cm × 13 cm × 3.5 cm. The pathology report endorsed malignant glands present in a background of necrosis (Figure 2). 

**Figure 3:**
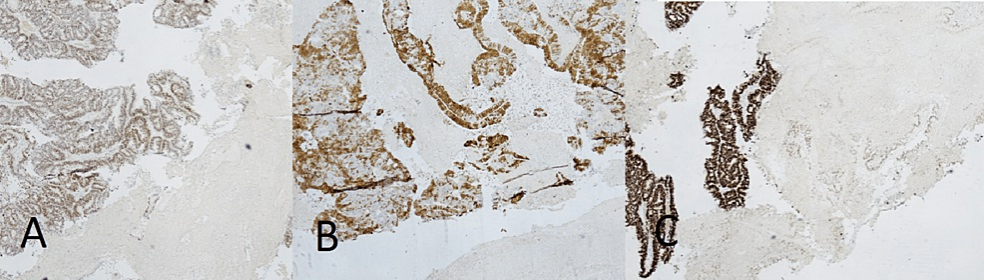
(A) PAX8 immunostain diffusely positive, (B) ER stain diffusely positive, and (C) p16 stain showing patchy positivity.

PAX8 and ER immunostaining were diffusely positive, while p16 staining was patchy. CDX2, CK7, and CK20 were negative (Figure 3). 

**Figure 4:**
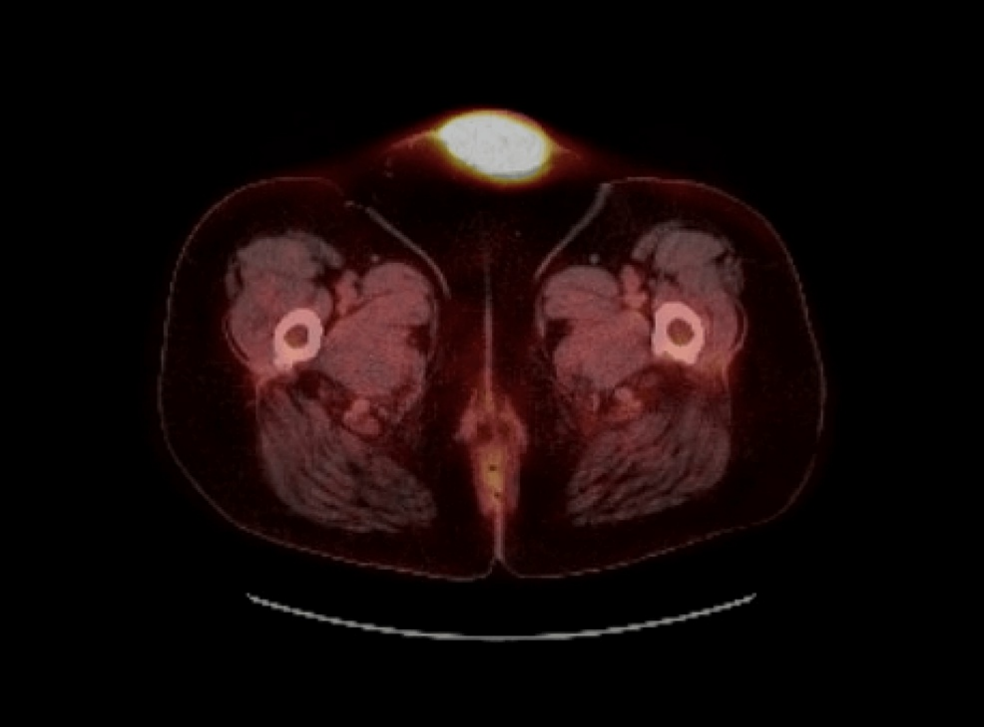
Transverse view of the CT-PET.

The diagnosis was noted to be adenocarcinoma favoring gynecological origin. A CT-PET scan was performed to rule out further metastatic disease, which was negative other than the known pubic soft tissue tumor (Figure 4).

The patient returned to the operating room for oncologic resection. While under general anesthesia and in the supine position, an elliptical incision was made surrounding the mass with 2 cm circumferential margins. The incision reached the subcutaneous fat, and the entire mass was removed and handed off as a specimen. A Jackson-Pratt drain (Cardinal Health, Waukegan, IL) was placed in the deep portions of the wound and brought out through the anterior abdominal fat. The fat and skin were reapproximated with 0 and 3-0 Vicryl sutures (Ethicon, LLC., San Lorenzo, PR) respectively. The final pathology of the lesion was concordant with the core biopsy, and all margins were negative. Given the R0 status and routine surveillance, an oncology consultation did not recommend any additional therapy.

## Discussion

After lung, breast, and colorectal cancer, endometrial carcinoma is the fourth most common cancer in women. It is also the most common gynecological cancer [3]. Sohaib et al. found that recurrent endometrial adenocarcinoma within two years of primary surgery was 64%, with the lymph nodes and vagina being the most likely spots of recurrence [4]. The cutaneous spread of this cancer is incredibly rare, with a reported prevalence of only 0.8% [2]. 

Morcellation, a minimally invasive technique that involves cutting the uterus into smaller pieces and removing it, has been widely stopped in the United States due to the risk of malignant seeding [5]. Scant surgical literature investigates the effects of an open hysterectomy approach and cancer seeding. In the reported cases, metastatic lesions are most commonly found at the initial surgery site. It is likely in this case that seeding did occur during the initial procedure, as the final pathology lends a diagnosis of adenocarcinoma favoring gynecological origin. 

Rarely, similar cases to this have been reported. The Indian Journal of Dermatology reported a case of a 50-year-old woman with fungating lesions on her vulva due to spread from her cervix [6]. El M’rabet et al. presented a case in 2012 of a 72-year-old woman with a history of adenocarcinoma and total abdominal hysterectomy that ended up having more than 20 metastatic lesions, both cutaneous and visceral [7]. Perhaps the most similar case to ours comes from Atallah et al., who reported a 62-year-old woman who underwent a total abdominal hysterectomy three years prior to developing a large mons pubis mass [8]. Of the three cases mentioned above, only one patient survived at the time of reporting. It is clear why close follow-up for cancer patients is indicated.

Due to the early diagnosis, high clinical suspicion, and surgical resection of this patient's malignancy, the patient continues to do well and has shown no evidence of disease recurrence or complications on follow-up.

## Conclusions

Soft tissue masses can oftentimes present with vague symptoms and can be misleading on examination. A thorough history with a focus on oncologic aspects is invaluable to suspicious appearing lesions. This case highlights that metastatic deposits in soft tissue can occur during open abdominal oncologic procedures and should remain in the differential diagnosis despite statistical rarity.
